# Encapsulation of Cyclosporine A-Loaded PLGA Nanospheres in Alginate Microbeads for Anti-Inflammatory Application

**DOI:** 10.1021/acsomega.3c08438

**Published:** 2024-02-01

**Authors:** Su Yee Win, Mongkol Chavalitsarot, Komgrit Eawsakul, Tassanee Ongtanasup, Norased Nasongkla

**Affiliations:** † Department of Biomedical Engineering, Faculty of Engineering, Mahidol University, Nakhon Pathom 73170, Thailand; ‡ Thailand Research Fund through the Royal Golden Jubilee Ph.D. Program, Phayathai, Bangkok 10400, Thailand; § Thailand International Cooperation Agency (TICA), Thungsonghong Laksi District, Bangkok 10210, Thailand; ∥ Department of Applied Thai Traditional Medicine, School of Medicine, Walailak University, Nakhon Si Thammarat 80160, Thailand

## Abstract

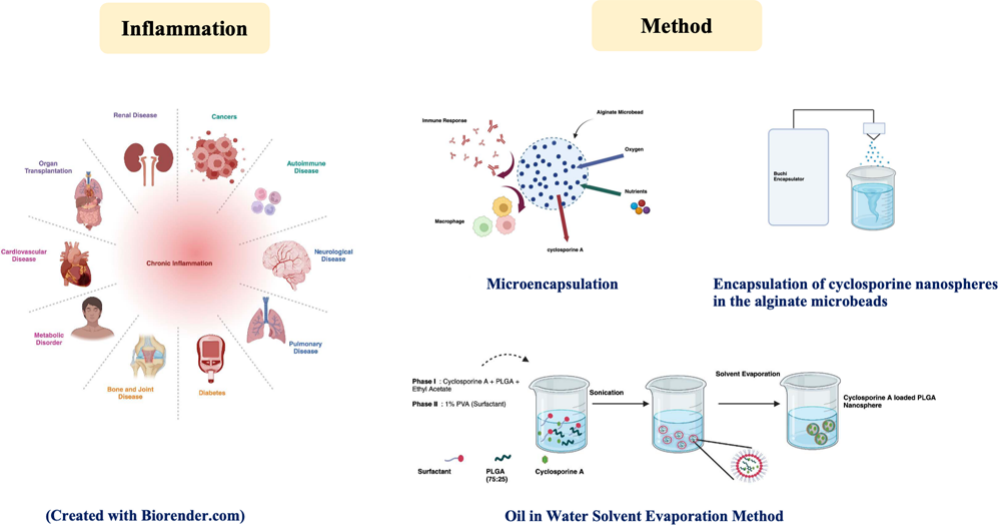

The controlled release of cyclosporine A (CsA) microencapsulated in alginate microbeads is a novel drug delivery system for the treatment of inflammatory diseases. In this study, CsA-loaded nanospheres encapsulated in alginate microbeads were applied to evaluate their controlled release profile and anti-inflammatory activity. Initially, a controlled-release drug delivery system was created by encapsulating CsA-loaded PLGA nanospheres within alginate microbeads. CsA-loaded PLGA nanospheres had a diameter of 418.70 ± 59.08 nm, a zeta potential of −22 ± 0.57 mV, and a polydispersity index of 0.517 ± 0.010. CsA-loaded nanosphere-encapsulated alginate microbeads were stable for 37 days. After encapsulating CsA-loaded PLGA nanospheres in the alginate microbeads, 5.60% of CsA was released after 24 h, and approximately 85.90% of the drugs were diffused until day 64. The cytotoxic and anti-inflammatory properties of the CsA released from the microbeads were evaluated in vitro using a murine macrophage cell line (RAW 264.7 cells). CsA-loaded nanosphere-encapsulated alginate microbeads inhibited 39.47 ± 1.71% of nitric oxide production from the RAW 264.7 cells on day 3, whereas nanosphere-encapsulated alginate microbeads inhibited 18.45 ± 1.56% only. CsA released from CsA-loaded nanosphere-encapsulated alginate microbeads had a RAW cell viability of 82.73 ± 5.58% on day 3 compared to 87.59 ± 0.69% of nanosphere-encapsulated alginate microbeads. The efficacy of the CsA-loaded nanosphere-encapsulated alginate microbeads in protecting the immune system via a controlled drug delivery system was established through anti-inflammatory and cell viability evaluation. Based on this research, the controlled release of CsA-loaded nanosphere-encapsulated alginate microbeads provides an innovative treatment for inflammatory diseases.

## 1 Introduction

Cyclosporine A (CsA) is a cyclic polypeptide consisting of 11 amino acids used to treat various diseases, including psoriasis, uveitis, transplant rejection, and inflammatory diseases.^1^ It is the first immunosuppressant drug and controls the IFN-g/STAT1 and IL-4/STAT6 signaling pathways to regulate the polarization of M1 and M2 macrophages in inflammatory reaction.^2−4^

Most cyclosporine formulations are administered orally. It has a poor pharmacokinetic profile, insufficient delivery to anti-inflammatory target tissues, and severe side effects. Due to its hydrophobicity, poor bioavailability, high molecular weight (1203 Da), and low permeability, oral CsA is incapable of maintaining steady blood levels of the drug. After administration, oral CsA is metabolized by the Cytochrome P450 3A4 enzyme in the liver and excreted via the bile. Long-term exposure to the CsA metabolites causes irreversible renal damage.^5,6^ In this regard, new formulation approaches, such as nanotechnology, have enhanced CsA delivery, improved therapeutic efficacy, and overcome poor bioavailability and water solubility of CsA.^7^ In addition, the use of drug-encapsulated microbeads to protect drugs against biological conditions such as pH, enzymes, and the immune system is an innovative approach to reducing the limitation of CsA.^8,9^

In this study, alginate microbeads with a controlled drug delivery system aim to increase the drug concentration at the site of action and to extend the therapeutic effect of a drug by releasing it continuously after a single dose. Moreover, alginate microbeads can be injected directly into the intraperitoneal route for acute liver failure treatment.^10−12^

The local CsA administration permits targeted immunosuppression at a specific site or organ, which can be especially advantageous when systemic immunosuppression is undesirable or increases the risk of adverse effects. As a result, the local administration of CsA minimizes systemic exposure to CsA while achieving immunosuppression at the intended site. Typically, a lower dose of CsA is required when the drug is administered locally compared to systematically.

Moreover, local drug delivery can increase drug concentrations at the site of action and improve the drug’s efficacy in treating localized disorders. Therefore, the local drug delivery method must accumulate significant quantities of CsA at the targeted site of action and prevent its distribution to adjacent organs. To avoid an immune response to implants or transplanted tissues, local administration of CsA maintains therapeutic levels in the target tissue for extended periods.^13^

In biomedical applications, injectable alginate microbeads that can deliver effective drugs locally are emerging as an alternative to conventional oral dosage forms.^8,9,14,15^ Alginate is the most used polysaccharide for cell encapsulation technology to produce alginate microbeads with a semipermeable membrane that permits the exchange of nutrients, oxygen, and drug metabolites through the pores of the microbeads.^16−18^ Alginate microbeads can be produced using ionotropic gelation, cross-linking, emulsion gelation, spray drying, and simple and complex coacervation phase separation methods.^19−22^ Due to sodium alginate’s biocompatibility, hydrophilicity, biodegradability, and nontoxicity, it is used in the controlled-release drug delivery system.^23−26^

**Figure 1:**
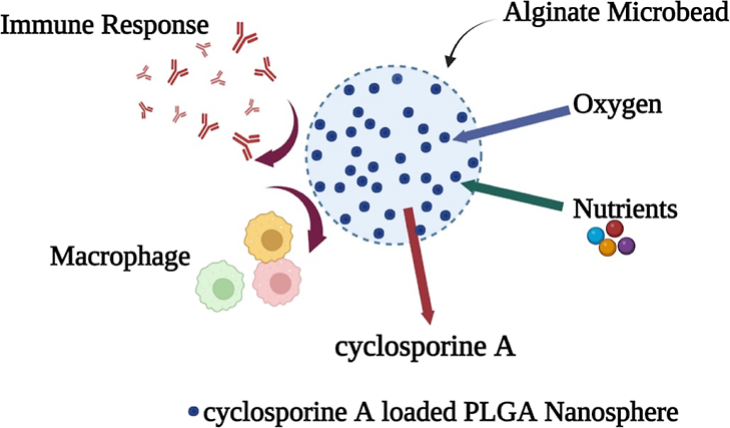
Encapsulation of a CsA-loaded PLGA nanosphere in an alginate microbead (created with Biorender.com).

In this work, in Figure 1, CsA was initially encapsulated in the PLGA (75:25) nanospheres using an oil in water emulsion. The nanospheres can control loaded drug release by altering the copolymer ratios.^27^ The biodegradable alginate microbeads with CsA-loaded PLGA nanosphere encapsulation were produced by cross-linking anionic polysaccharide sodium alginate with divalent cations (Ca^2+^) from a calcium chloride solution. After the fabrication of CsA-loaded nanosphere-encapsulated alginate microbeads, the morphology and stability of the microbeads and the release amount, anti-inflammatory activity, and cell viability of the drug released from the microbeads were evaluated by using the murine macrophage cell line (RAW 264.7 cells).

## 2 Materials and Methods

### 2.1 Materials

Sodium alginate was purchased from Buchi, Ireland. Calcium chloride was purchased from Sigma-Aldrich (St. Louis, MO, USA). CsA was purchased from LC Laboratories, USA. PLGA (75:25; *M*_w_ = 31 kDa) was purchased from NanoPolyPEG Co., Ltd. Ethyl acetate (Laboratory Plus) was from Honeywell International Inc., USA. Poly(vinyl alcohol) (PVA) (average *M*_w_ = 13–23 kDa, 98% hydrolyzed) was from Sigma-Aldrich (St. Louis, MO, USA). CaCl_2_ was obtained from the EMD Millipore Corporation in the United States. HEPES (Bioperformance Certified, ≥99.5%) was from Sigma-Aldrich (St. Louis, MO, USA). DMEM-F12 was purchased from Biowest, France. Glutamax 100X, penicillin–streptomycin, 0.125% trypsin–EDTA, and fetal bovine serum were purchased from Gibco in the United States. Disodium hydrogen phosphate anhydrous (Na_2_HPO_4_) and sodium chloride (NaCl) were purchased from RCI Labscan Limited (Bangkok, Thailand). The RAW 264.7 cell line was obtained from the American Type Culture Collection (ATCC). The lipopolysaccharide (LPS) was purchased from Merck (Germany). The Griess reagent was purchased from Sigma Chemical Co. (St. Louis, MO, USA) and was prepared by dissolving 1% sulphanilamide and 0.1% *N*-(l-naphthyl)-ethylenediamine dihydrochloride in 2.5% H_3_PO_4_.

### 2.2 Preparation of CsA-Loaded PLGA Nanospheres

CsA-loaded PLGA nanospheres (CsA-NPs) were produced by using an oil-in-water emulsion diffusion evaporation technique. CsA and PLGA (75:25 copolymer ratio, 22 kDa molecular weight) were dissolved in ethyl acetate and sonicated at 80% amplitude for 30 s with a sonicator (Sonics Vibra-Cell Ultrasonic Processor) to ensure complete dissolving. The organic phase was added dropwise to 20 mL of an aqueous phase containing 1% PVA solution to prepare the primary emulsion. The mixture was sonicated at an amplitude of 80% for 3 min. The nanoemulsion was introduced drop-by-drop to a 1% PVA solution for 6 min with an 80% amplitude. The nanoemulsion was evaporated overnight to eliminate the harmful effects of the organic solvent. The nanosphere suspension was centrifuged at 3000 rpm for 2 min to eliminate large nanospheres. The nanosphere suspension was centrifuged for 45 min at 14,000 rpm and 4 °C to remove the remaining PVA solution. The nanosphere was rinsed with distilled water three times to eliminate excess surfactant and PLGA residue. After being purified, the nanosphere pellets were resuspended in 5 mL of deionized water. To estimate the total dry weight of the yield nanosphere, 1 mL of nanosphere suspension was freeze-dried at −45 °C and then stored between 4 and 8 °C. It has been proposed that freeze-drying can improve the long-term stability of colloidal nanoparticles. The stability of nanoparticles in aqueous media is a crucial obstacle to their application. The freeze-drying process assures the long-term stability and preservation of pharmaceutical and biological products.^28^ Before the nanospheres were encapsulated in alginate microbeads, their particle size distribution, zeta potential, and polydispersity index were analyzed.^29^

### 2.3 Characterization of CsA-Loaded PLGA Nanospheres

#### 2.3.1 Assessment of the Size Distribution and Zeta Potential of CsA-Loaded PLGA Nanospheres

The particle size distribution, zeta potential, and polydispersity index (PDI) of nanospheres were determined by using dynamic light scattering (Zetasizer Nano ZS, Malvern, UK). After diluting 100 μL of nanosphere suspension with 900 μL of deionized water, the zeta potential was calculated by measuring the electrophoretic mobility of the nanospheres in an electric field. At 25 °C, the particle size distribution of nanospheres in deionized water was determined by analyzing scattered light. The data for each sample were determined in triplicate samples.^29^

#### 2.3.2 Evaluation of Yield, Drug Loading Capacity, and Entrapment Efficiency of CsA-Loaded PLGA Nanospheres

High-performance liquid chromatography(HPLC) was used to determine the CsA-loading concentration in the PLGA nanospheres (HPLC, Dionex Ultimate 3000). Five mg of freeze-dried nanospheres was dissolved in 25 mL of a solution containing 70% acetonitrile and 30% distilled water. This solution was used as the mobile phase with a flow rate of 1 mL/min to determine the CsA loading content in the nanosphere. The HPLC column was a C_18_ (isocratic mode) column with a 4.6 mm inner diameter, 100 mm length, 3.5 m particle size, and 60 °C temperature. At 210 nm, the absorbance of known concentrations of CsA was measured to establish a standard curve. The yield percentage, drug loading content (DL), and encapsulation efficiency (EE) of CsA nanospheres were determined using eqs 1–3.^30−32^
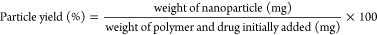
1
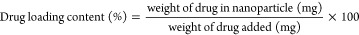
2
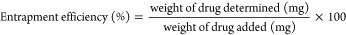
3

#### 2.3.3 Assessment of the Morphology of CsA-Loaded PLGA Nanospheres

To evaluate the morphology of the nanospheres, freeze-dried powders of PLGA nanospheres and CsA-loaded PLGA nanospheres were coated with platinum–palladium and placed on a grid. The nanospheres were examined using a scanning electron microscope and an energy dispersive X-ray spectrometer–SEM–EDS (JEOL, JSM-IT500HR). The SEM images demonstrate the nanosphere’s smooth surface, a few islands, and drug aggregation on its surface.^33−35^

#### 2.3.4 Release Study of CsA-Loaded PLGA Nanospheres

Before the nanospheres were encapsulated in an alginate microbead, CsA released from the nanospheres was studied to develop a controlled drug delivery system. Using a dialysis membrane with a molecular weight cutoff of 15 kDa composed of regenerated cellulose (Sigma-Aldrich, USA), the controlled release patterns of CsA from PLGA nanospheres were investigated. The dialysis membrane was washed with distilled water before use. After centrifuging the nanosphere, the nanosphere pellet containing the entrapped drug (130 mg of cyclosporine was entrapped in the nanospheres) was collected and dispersed in 1 mL of a released solution containing phosphate buffer saline and 0.05% w/v of nonionic surfactant Tween 80 at pH 7.4.^36^ The 10 mL of released medium was placed outside the membrane. The drug release from PLGA nanospheres was studied in an incubator (Wisecube, Fuzzy Control System, Witeg Laboratory Instruments) with a shaking speed of 90 rpm and a temperature of 37 °C. At predetermined intervals, 1 mL of released solution was collected, and 1 mL of medium was added to the outside of the membrane. Using HPLC (Dionex Ultimate 3000), we determined the amount of CsA released from the nanospheres. The amount of CsA loaded in the nanospheres was used to calculate the CsA release (ug) in the medium, which was then determined as a percentage of the total CsA release.^29,30^

### 2.4 Encapsulation of CsA-Loaded PLGA Nanospheres in Alginate Microbeads

Before fabricating drug-loaded microbeads with encapsulator B-395 (BUCHI, Ireland), a CsA-loaded PLGA nanosphere suspension containing 0.9% sodium chloride was sterilized in a biosafety cabinet under UV light for 1 h. CsA is sterilized using UV irradiation for 1 h.^37^ In a dry autoclaved bottle, sodium alginate was dissolved in 0.9% normal saline using continuous stirring. In a biosafety cabinet, the alginate solution was sterilized by using a 0.2 μm syringe filter. The UV-sterilized CsA nanosphere suspension was then carefully mixed with 3% alginate solution to achieve a final concentration of 25 mg/mL (25 mg of CsA nanospheres in 1 mL of 1.5% alginate solution). The BUCHI encapsulator was set to 700 Hz and 2.2 kV with a flow rate of 7.5 mL/min. A CsA nanosphere-loaded alginate microbead was created by extruding the nanosphere-containing alginate solution via a 300 μm nozzle of encapsulator into a solution of 115 mM CaCl_2_ and stirring it for 15 min to produce CsA-loaded nanosphere-encapsulated alginate microbeads.^12^

### 2.5 Release Study of CsA from Alginate Microbeads

Using a regenerated cellulose dialysis membrane with a molecular weight cutoff of 15 kDa (Sigma-Aldrich, USA), the controlled release patterns of CsA from alginate microbeads were investigated. CsA-loaded nanosphere-encapsulated alginate microbeads (1500 mg of CsA was entrapped in the microbeads) were collected and distributed in 10 mL of a release solution containing phosphate buffer saline and 0.05% (w/v) Tween 80 at pH 7.4. Before use, the membrane was washed with distilled water. The dialysis bag was immersed in a volume of 1000 mL of the release medium. At periodical intervals, 1 mL of released solution was collected, and a new 1 mL was added to the exterior of the dialysis membrane. The drug release studies were conducted at 37 °C and 90 rpm in an incubator shaker (Wisecube, Fuzzy Control System, Witeg Laboratory Instruments). Using HPLC (Dionex Ultimate 3000), we determined the amount of CsA released from the microbeads. The amount of CsA loaded in the nanospheres was used to calculate the CsA release (μg) in the medium, which was then determined as a percentage of the total CsA release from the alginate microbeads.^29,30^ After that, the controlled drug release percentage and time from the nanosphere and alginate microbead were then compared.

### 2.6 Assessment of Drug Release Kinetics

The release kinetic of CsA released from PLGA nanospheres and alginate microbeads was calculated using the following eq 4
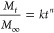
4*k* is a kinetic constant associated with the hydrogel system and *n* represents the diffusion exponent, which represents the hydrogel samples’ drug transport mechanism. *M*_*t*_ and *M*_∞_ represent the cumulative CsA release at time t and the release equilibrium time, respectively. The values of n are determined by the slope of ln(*M*_*t*_/*M*_∞_) versus ln(*t*). If n is greater than 1.0, polymer chain relaxation becomes the rate-controlling factor for a relaxation or swelling controlled-release mechanism. A value of *n* < 0.5 corresponds to a pure Fickian diffusion mechanism. The value of *n* between 0.5 and 1.0 indicates a non-Fickian diffusion mechanism, whereas the system was characterized by Fickian diffusion and relaxation-controlled release.^38^

### 2.7 Assessment of the Morphology of CsA-Loaded Nanosphere-Encapsulated Alginate Microbeads

To investigate the morphology of the microbeads, two different types of microbeads were prepared: alginate microbeads and CsA-loaded nanosphere-encapsulated alginate microbeads. The microbeads were then treated with 3% glutaraldehyde for 5 min. After removing glutaraldehyde, samples were dehydrated for a further 10 min in each of the five ethanol solutions that had been serially diluted to 30, 50, 70, 90, and 100%. The samples were then treated for 5 min in 1 mL of hexamethyldisilane. The samples were placed in a desiccator and dried at room temperature for 24 h. The samples were then placed on a grid and coated with platinum and palladium. The beads were examined with a scanning electron microscope and an energy-dispersive X-ray spectrometer—EDS (JEOL, JSM-IT500HR). The SEM photos indicate the smooth surfaces of alginate microbeads and a few islands and drug aggregation on the surface of the CsA-loaded nanosphere-encapsulated alginate microbeads.^33,39^

### 2.8 Stability Testing of Microbeads

Initially, two different types of microbeads, alginate microbeads and CsA-loaded nanosphere-encapsulated alginate microbeads, were produced using an Encapsulator B-395 Pro in the same setting. After the microbeads were suspended in DMEM in a six-well plate, they were incubated at 37 °C with 5% CO_2_ in a CO_2_ incubator. The microbeads’ morphology was inspected until broken, and then ImageJ was used to calculate their sizes.^12^

### 2.9 Anti-inflammatory Activity of CsA Released from the Alginate Microbeads

#### 2.9.1 Induction of Raw Cells with LPS

Following a 24 h incubation period, the DMEM was removed from the 96-well plate containing RAW 264.7 cells. To maximize the effects of LPS induction, the cells were treated with LPS at concentrations of 1, 3, and 5 μg/mL. It was subsequently determined that the optimal concentration of LPS (1 μg/mL) was effective in inducing raw cells, as demonstrated by its relatively high induction rate to the RAW cells in comparison to the other concentrations.

#### 2.9.2 Determination of Nitric Oxide Concentration

The anti-inflammatory activities of CsA released from the alginate microbeads were investigated with the murine macrophage cell line (RAW 264.7 cells). In this study, the nanosphere-encapsulated alginate microbeads were used as control microbeads, and the CsA-loaded nanosphere-encapsulated alginate microbeads were used as study microbeads. First, 1 × 10^5^ RAW 264.7 cells per well were seeded in a 96-well plate and allowed to adhere to the plate surface for 24 h. To induce the RAW 264.7 cells, 100 μL of LPS (1 μg/mL) was added to each well of the incubated well plate after incubation. LPS stimulation initiated the production of nitrite by the cells in the medium. The activated RAW 264.7 cells were treated with CsA released from the alginate microbeads after 3 h, 6 h, 12 h, 24 h, and day 3.

Griess reagent was used to detect the concentration of nitrite, the final product of nitric oxide (NO) metabolism in the inflammatory response. After mixing 100 μL of culture supernatants with 100 μL of Griess reagent for 10 min, the nitrite concentration in the culture supernatants was measured at an absorbance of 570 nm. The percentage of nitric oxide inhibition was then computed in eq 5 to establish the anti-inflammatory effect of CsA released from the alginate microbeads. Experiments were performed in triplicate, and the results of the study were reported as means with standard deviations.^40^
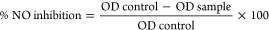
5

### 2.10 Cell Viability of CsA Released from the Alginate Microbeads

MTT assay was used to determine in vitro cell viability of the released CsA from the alginate microbeads against the macrophage cell line (RAW 264.7). In this study, the RAW cells treated without LPS and the cells treated with LPS were used as controls. First, 1 × 10^5^ RAW 264.7 cells per well were seeded in a 96-well plate and allowed to adhere to the plate surface for 24 h. After incubating, 100 μL of LPS (1 μg/mL) was added to each well of the incubated well plate to induce the RAW cells. The activated RAW 264.7 cells were treated with CsA released from the microbeads after 3 h, 6 h, 12 h, 24 h, and day 3. After incubating for 24 h, the supernatant was removed from the wells, and 50 μL of MTT (2 mg/mL in serum-free culture media) was added to each well. Plates were incubated at 37 °C for 90 min. After carefully removing the MTT solution, 200 μL of DMSO was added and thoroughly mixed for 30 min; the purple-colored formazan was produced by the living cells. At 570 nm, the absorbance of the sample was measured using a microplate reader (Lab Systems Multiskan RC, USA). The formula in the equation expressed the viable cell proportion as a percentage. Experiments were conducted in triplicate, and study results were provided as means with standard deviations.^41^
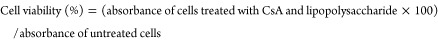
6

## 3 Results and Discussion

### 3.1 Characterization of CsA-Loaded PLGA Nanospheres

#### 3.1.1 Assessment of CsA-Loaded PLGA Nanospheres

The stability of nanospheres was examined based on their size distribution, polydispersity index, shape, and surface charges, after their preparation.^42^ It has been found that the preparation procedure affects the size of polymeric nanoparticles. The sonication process produces nanospheres that are smaller than the homogenization method. As the sonication speed increased, the size of the nanosphere decreased.^31^ A zeta sizer was applied to analyze the nanospheres’ size distribution. The difference in electrokinetic potential between the dispersion medium and the fluid’s stationary layer indicates the surface charges of the nanospheres. It was reported in Table 1 that the nanoparticles showed a negative zeta potential ranging from −16 to −35 mV. The negative zeta potential of the nanospheres indicates that they do not interact with the cell surface, which also has a negative zeta potential; as a result, the nanospheres do not interfere with the viability of the cell inside the human body.^43^ Polydispersity index was used to measure the homogeneity of a colloidal solution. The PDI varies between 0.0 and 1.0.^29,44^ A high PDI score suggests a sample with a population of varying sizes that is highly polydisperse. The nanosphere suspension has a relatively narrow particle size distribution if the PDI is less than 0.5.^45−47^

**Table 1:** Particle Size Distribution and Zeta Potential of CsA-Loaded PLGA Nanospheres

nanosphere	size (nanometer)	zeta potential (mV)	PDI
PLGA nanosphere	411.00 ± 42.46	–29 ± 0.51	0.446 ± 0.021
CsA-loaded PLGA nanospheres	418.70 ± 59.08	–22 ± 0.57	0.517 ± 0.091

**Figure 2:**
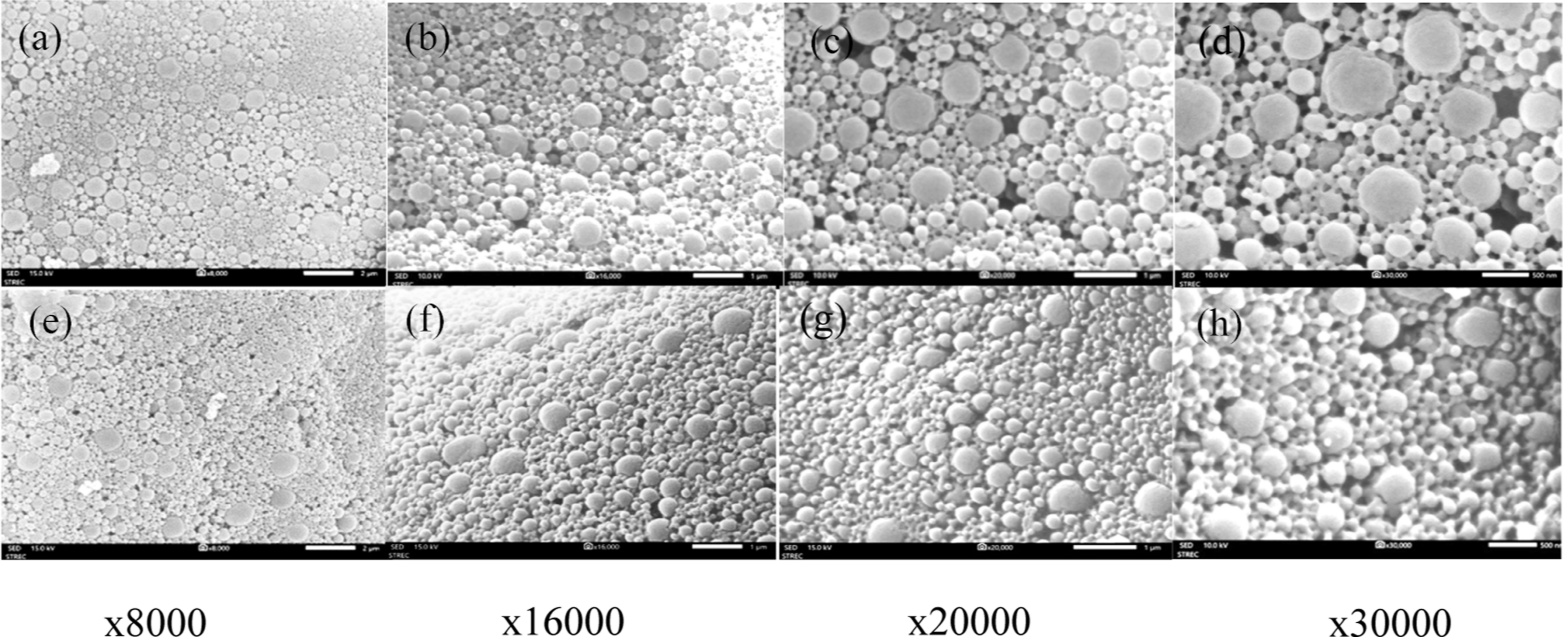
SEM image of PLGA nanospheres (a–d) and CsA-loaded PLGA nanospheres (e–h).

In this study, the CsA-loaded PLGA nanospheres with the desired size were produced using probe sonication. In Table 1, the PLGA bare nanospheres had a mean size distribution of 411 ± 42.46 nm in diameter, a zeta potential of −29 ± 0.51 mV, and a PDI of 0.446 ± 0.021. CsA-loaded PLGA nanospheres had a diameter of 418.70 ± 59.08 nm, a zeta potential of −22 ± 0.57 mV, and a PDI of 0.517 ± 0.01. Figure 2 represents spherical nanospheres with a smooth surface as captured by scanning electron microscopy (SEM). The particle size of the nanospheres is well correlated with the zeta-sizer and SEM measurements. The drug loading content of CsA-loaded PLGA nanospheres was 10.72 ± 0.81%, while the entrapment efficiency was 48.58 ± 1.72%. The production yield of nanospheres was 75.74 ± 4.52%. CsA was a very poorly water-soluble drug; during preparation, a small amount of the drug was lost to the aqueous phase.

#### 3.1.2 Assessment of the Morphology of CsA-Loaded PLGA Nanospheres

The morphology of the nanospheres was characterized by spreading freeze-dried PLGA nanospheres and CsA-loaded PLGA nanospheres on a grid, drying them in a vacuum, and coating them with platinum–palladium for 15 min. The coated specimens were examined by a scanning electron microscope and energy dispersive X-ray spectrometer–SEM–EDS (JEOL JSM-IT500HR). ImageJ software calculated the nanosphere’s particle size using the scale bar. Each sample’s information was evaluated in triplicate.^31,45^ In this work, the particle size of the PLGA nanosphere, as determined by a scanning electron microscope, was 384.09 ± 18.24 nm, and that of the CsA nanosphere was 398.66 ± 7.4 nm in Figure 2.

### 3.2 Characterization of CsA Nanosphere-Encapsulated Alginate Microbeads

#### 3.2.1 Release Study of CsA-Loaded PLGA Nanospheres and CsA-Loaded Nanosphere-Encapsulated Alginate Microbeads

The dialysis membrane method carried out the CsA release from the nanospheres and alginate microbeads. PLGA (75:25), the polymer used in the preparation, has a more significant proportion of hydrophobic methyl groups in the LA ratio, which may slow the degradation of the drug-polymer matrix (Nasongkla, 2009). In this work, encapsulating a CsA nanosphere in an alginate microbead is a proposed approach for preventing subtherapeutic or toxic after the administration of CsA. Before encapsulating CsA-loaded PLGA nanospheres into the alginate microbead, the drug release profile of the nanospheres was evaluated. They were then encapsulated in alginate microbeads to maintain the release of CsA within a therapeutic window for a few weeks and to suppress the immune response of the transplanted organs of the patient. This method eliminates the disadvantages of CsA, including poor bioavailability, hydrophobicity, low permeability through biological barriers, dose-dependent nephrotoxicity, lower drug physicochemical stability, and systemic toxicity.

The CsA drug release pattern from the nanospheres and the microbeads was a zero-order drug release system that releases the drug at a constant rate, and the drug release rate is independent of the concentration or the amount of drug that remains in the delivery system. The drug release profile was at a constant rate, and the cumulative drug release occurred over time.

**Figure 3:**
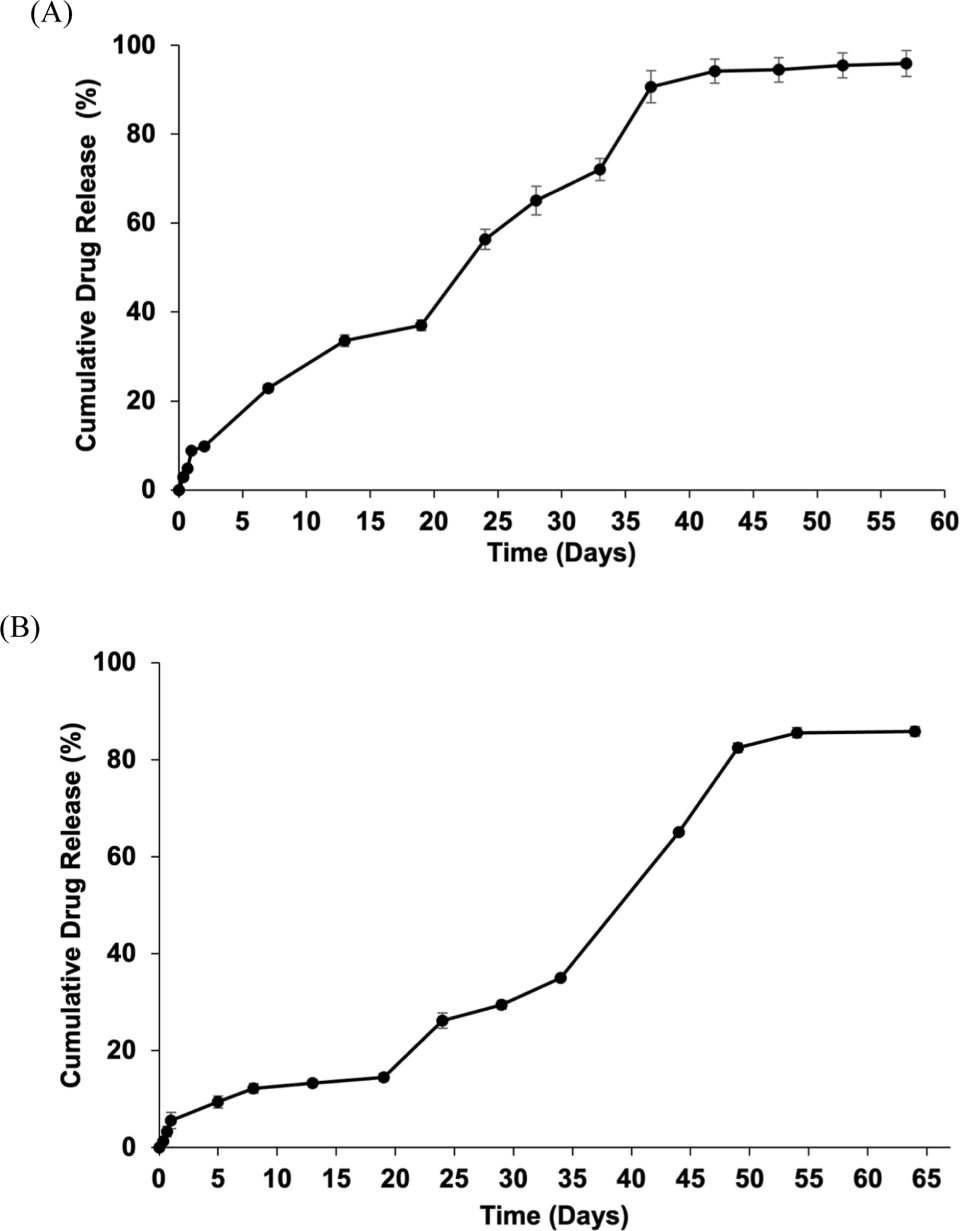
In vitro drug release studies of CsA (A) from the PLGA nanosphere (B) from alginate microbeads.

First, the CsA-loaded PLGA nanosphere’s drug release profile can be divided into three phases. In phase I, a burst release occurred due to the release of the unloaded drug from the nanosphere’s surface within 24 h of the drug release study. In phase II, drug diffusion through the polymer’s pores occurred slowly from day 1 to day 20. Due to the degradation activity of PLGA, there was substantial erosion of the CsA in phase III from day 20 to day 57 in the nanosphere and from day 20 to day 64 in the microbead. Figure 3A illustrates the triphasic release pattern of CsA from the nanosphere with a zero-order kinetic, whereas Figure 3B illustrates the CsA release from the alginate microbead. A burst-like release was detected from the nanosphere and microbeads within 24 h, followed by an increased drug release rate. The release profiles of CsA from CsA-loaded PLGA nanospheres and alginate microbeads show burst release within 24 h, followed by zero-order release kinetics (drug diffusion) from day 1 to day 20. Compared to a nanosphere-based drug delivery system, nanoparticles encapsulated in the alginate microbeads can control the drug release rate, which is approximately two times slower.

After day 20, the CsA release rate was increased in both the nanosphere and microbead due to the degradation of PLGA. CsA release was detected up to day 57 from the nanosphere and day 64 from the microbead. To treat acute liver failure, the CsA released by the alginate microbead is only necessary for 14 days.^12^

#### 3.2.2 Release Kinetics of CsA-Loaded PLGA Nanospheres and CsA-Loaded Nanospheres Encapsulated in Alginate Microbeads

**Figure 4:**
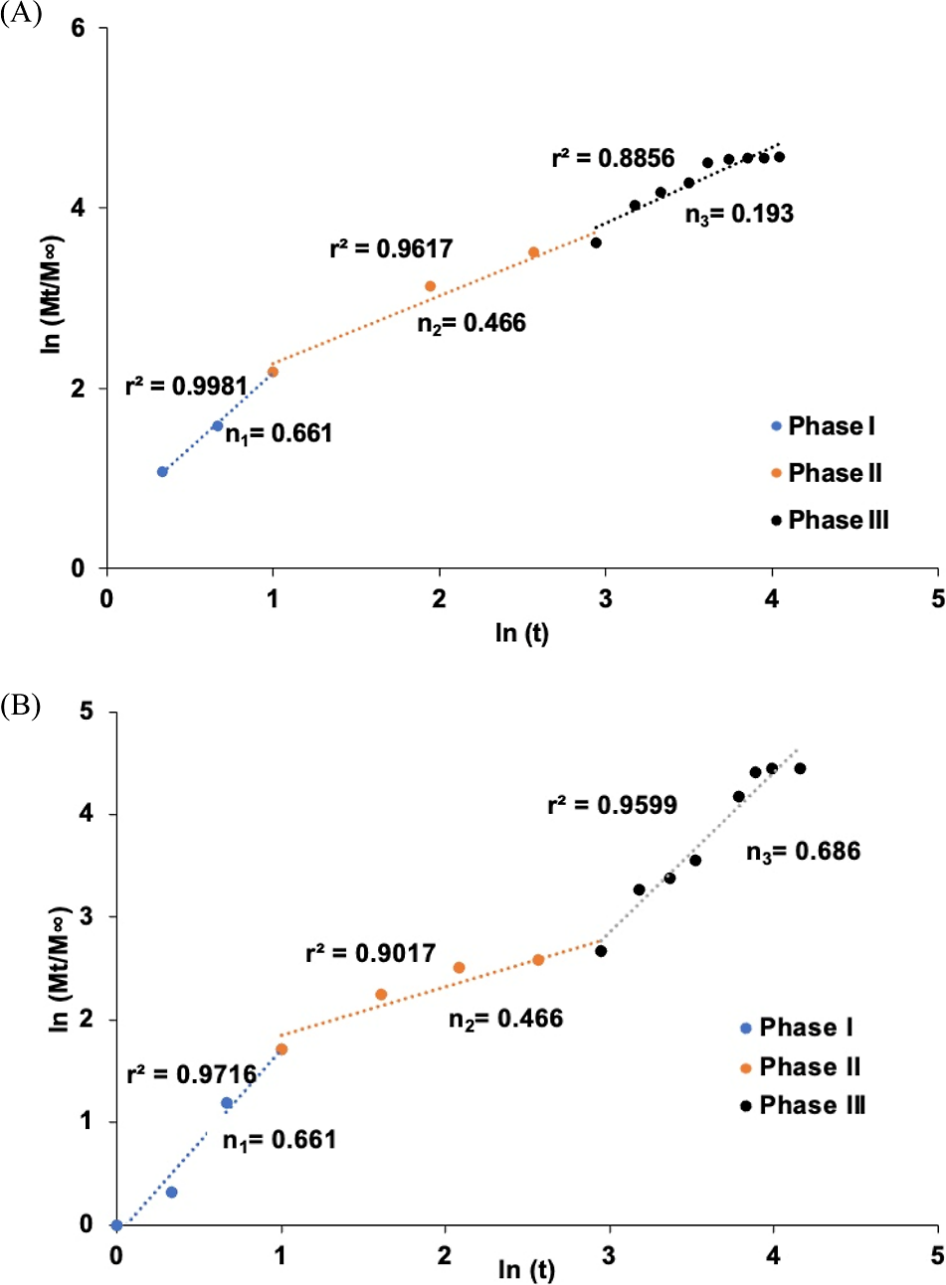
Plots of ln(*M*_*t*_/*M*_∞_) vs ln(*t*) for (A) CsA released from PLGA nanospheres and (B) CsA released from alginate microbeads.

In the release kinetic of CsA release from the nanosphere (Korsmeyer–Peppas model), in phase I of the CsA nanosphere release study in Figure 4A, the initial release showed *n*_1_ = 0.661 (*r*^2^ = 0.9981), and a burst release with non Fickian diffusion occurred due to the release of the unloaded drug from the nanosphere’s surface within 24 h of the drug release study. In phase II, however, *n*_2_ = 0.466 (*r*^2^ = 0.9617), which represents Fickian diffusion, the release of the drug steadily decreased because of the drug diffusing through the pores of the polymer. In phase III of the CsA nanosphere release study, due to the degradation activity of PLGA, there was a significant erosion of the CsA with *n*_3_ = 0.193 (*r*^2^ = 0.8855), indicating relaxation-controlled release of the CsA-loaded PLGA nanosphere.

In phase I of the CsA-loaded nanospheres encapsulated in alginate microbeads’ release study in Figure 4B, the initial release of CsA from the alginate microbeads (Korsmeyer–Peppa model) with non-Fickian diffusion demonstrated *n*_1_ = 0.661% (*r*^2^ = 0.9716), and a burst release occurred within 24 h. In phase II, however, *n*_2_ = 0.466 (*r*^2^ = 0.9017), representing Fickian diffusion, the drug release gradually decreased as the drug diffused through the pores of the microbeads. Due to the degradation activity of PLGA within the alginate microbeads, there was a significant erosion of CsA with *n*_3_ = 0.686 (*r*^2^ = 0.9599) in phase III of the CsA release from the microbeads, indicating non-Fickian diffusion release of the CsA from the microbeads.^38,48^

#### 3.2.3 Assessment of the Morphology of CsA-Loaded Nanosphere-Encapsulated Alginate Microbeads

**Figure 5:**
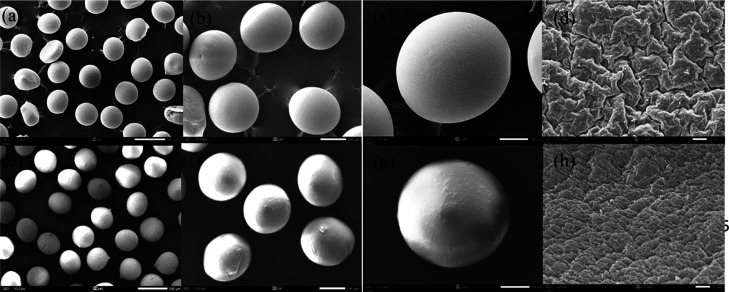
SEM image of alginate bare bead (a–d) and CsA-loaded nanospheres encapsulated in alginate microbeads (e–h).

Scanning electron microscopy (energy dispersive X-ray spectrometer–SEM–EDS) (JEOL JSM-IT500HR) was used to evaluate the morphology of bare alginate microbeads and CsA nanosphere-loaded alginate microbeads. First, the microbeads were initially fixed in ethanol, and their morphology was analyzed. The size of the alginate bare microbead was 388.72 ± 6.90 μm and that of the CsA-loaded nanosphere-encapsulated alginate microbeads was 399.27 ± 9.90 μm. SEM analysis in Figure 5 showed that CsA-loaded nanosphere-encapsulated alginate microbeads affected the surface morphology of microbeads. SEM images of alginate microbeads and CsA-loaded nanosphere-encapsulated alginate microbeads showed similar spherical shapes. In contrast to the smooth surface of alginate bare microbeads, CsA-encapsulated alginate microbeads showed a rough surface, probably due to the encapsulated nanospheres.^49^

#### 3.2.4 Stability Testing of Microbeads

**Figure 6:**
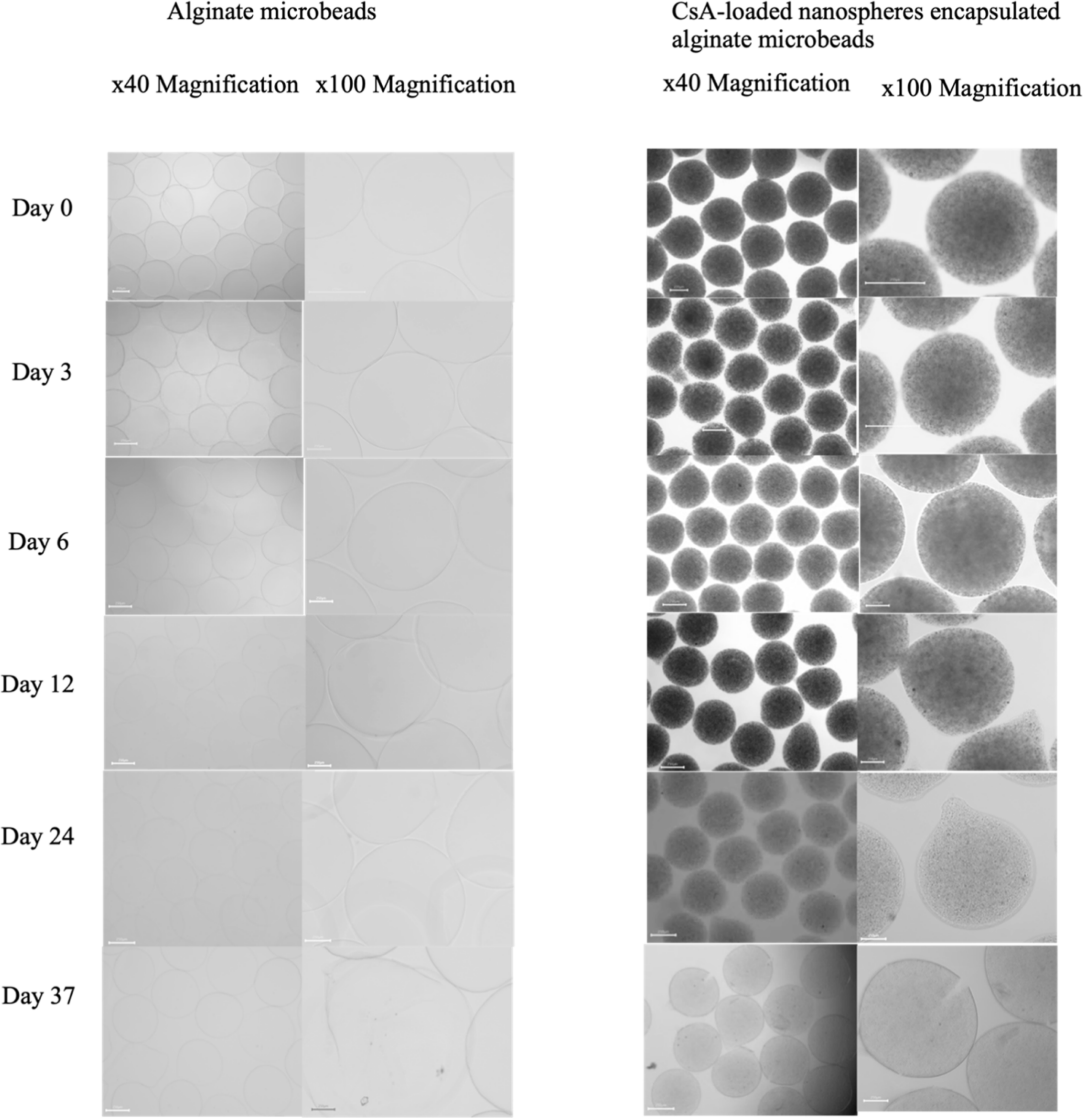
Photo series of microbead morphology at different observation times in DMEM/F-12. The scale bar is 250 μm.

**Figure 7:**
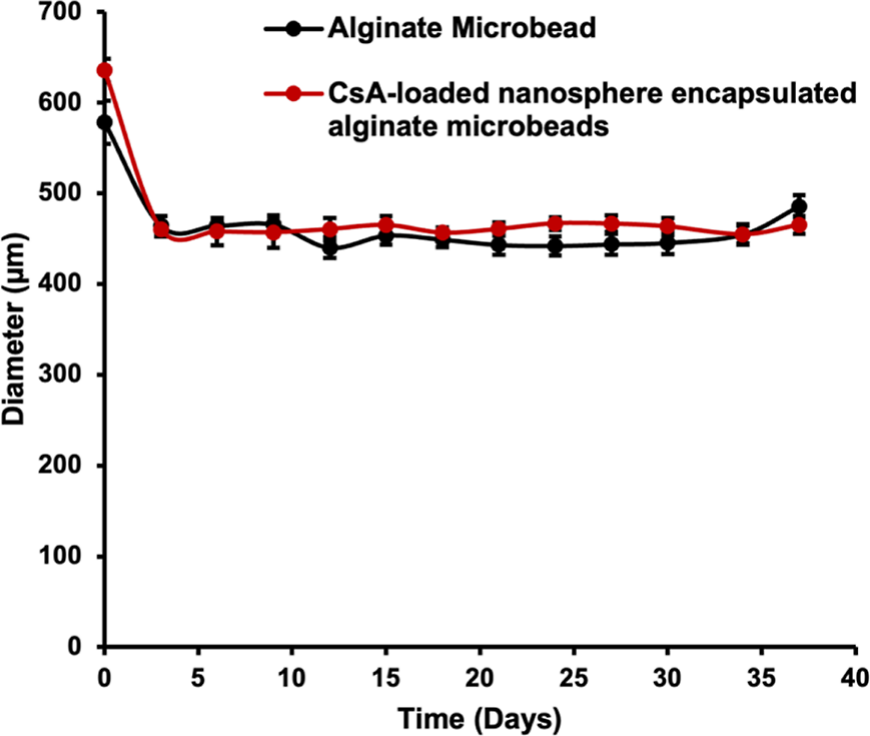
Microbead stability test.

The physical stability of the microbead is an essential factor in controlling the release of encapsulated drugs and the biological response after implantation. Alginate microbead deterioration is due to the chelating agent Ca^2+^ ions of the CaCl_2_ solution, which could induce the alginate beads to fuse during the gelation process.^50^ This study examined two types of microbeads in Figure 6, including alginate bare beads and CsA-loaded nanosphere-encapsulated alginate microbeads, for stability over time. In Figure 7, at day 0, the size of the alginate microbead was 578.65 ± 23.92 μm, and that of CsA-loaded nanosphere-encapsulated alginate microbeads was 635.75 ± 15.07 μm, respectively (*n* = 30). Microbeads had a spherical shape; however, alginate microbeads swelled faster than CsA-loaded nanosphere-encapsulated alginate microbeads; hence, they were broken more quickly in the DMEM. In addition, many alginate beads broke in a week after being produced.

Interestingly, the CsA-loaded nanosphere-encapsulated alginate microbeads did not expand. At day 12, some alginate and CsA-loaded nanosphere-encapsulated alginate microbeads began to change shape; however, CsA-loaded nanosphere-encapsulated alginate microbeads were significantly more spherical than unloaded alginate beads. After 37 days of incubation in DMEM, the size of the unloaded alginate microbead was 485.66 ± 12.38 μm, while the size of the CsA-loaded nanosphere-encapsulated alginate microbeads was 465.11 ± 9.0 μm. On day 37, the number of spherical CsA microbeads was more than that of alginate microbeads due to the swelling of alginate microbeads being more significant than that of CsA-loaded nanosphere-encapsulated alginate microbeads. In comparison to empty alginate microbeads, CsA-loaded nanosphere-encapsulated alginate microbeads were more stable.

Several in vivo studies have shown that alginate microbeads are stable in various tissue locations for more than 6 weeks. In vivo, oxidative, phagocytic, and enzymatic processes caused by cell response to the implanted material can accelerate biomaterial degradation. Calcium levels significantly influence the long-term stability of alginate microbeads in the microenvironment. In this study, the stability studies revealed that the beads remained physically and chemically stable for more than 45 days. The CsA released from the alginate microbead for treating acute liver failure is only necessary for 14 days.^12,50−52^

#### 3.2.5 Induction of Raw Cells with LPS

**Figure 8:**
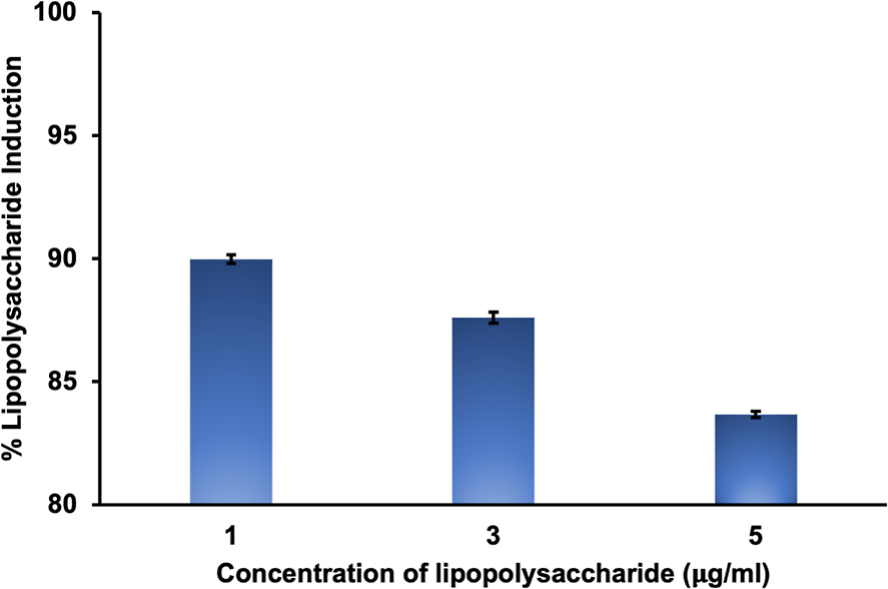
LPS induction on RAW 264.7 cells.

After 24 h of incubation, the DMEM was removed from the 96-well plate, including RAW 264.7 cells. To optimize the induction of LPS, the cells were exposed to treatment with different concentrations of LPS, such as 1, 3, and 5 μg/mL. The optimal LPS concentration (1 μg/mL) was subsequently determined to be effective in inducing raw cells because of its highest induction rate on the RAW cells compared to the other concentrations (Figure 8).

#### 3.2.6 Anti-inflammatory Activity of CsA Released from the Alginate Microbeads

The anti-inflammatory activities of CsA released from the alginate microbeads were investigated with a murine macrophage cell line (RAW 264.7 cells). In this study, the nanosphere-encapsulated alginate microbeads were used as control microbeads, and the CsA-loaded nanosphere-encapsulated alginate microbeads were used as study microbeads. First, 1 × 10^5^ RAW 264.7 cells per well were seeded in a 96-well plate and allowed to adhere to the plate surface for 24 h. To induce the RAW cells, 100 μL of LPS (1 μg/mL) was added to each well of the incubated well plate after incubation. LPS stimulation initiated the production of nitrite by the cells in the medium. The activated RAW 264.7 cells were treated with CsA released from the microbeads after 3 h, 6 h, 12 h, 24 h, and day 3.

**Figure 9:**
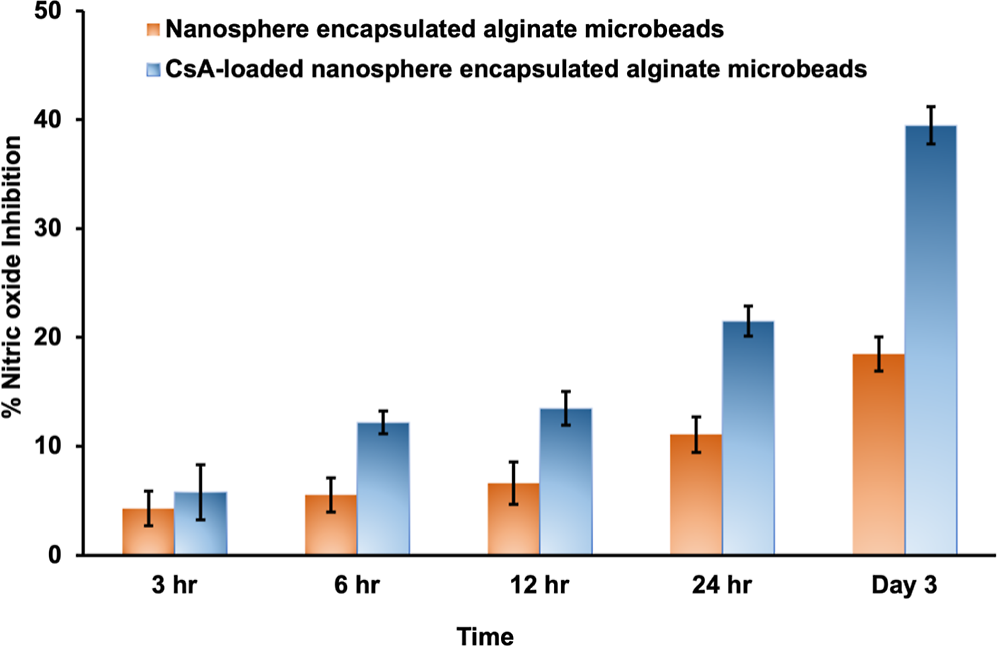
Anti-inflammatory activity of nanosphere-encapsulated alginate microbeads and CsA-loaded nanosphere-encapsulated alginate microbeads on RAW 264.7 cells.

In comparison to the control, nanosphere encapsulated alginate microbeads, the CsA-loaded nanospheres encapsulated alginate microbeads had 39.47 ± 1.71% nitric oxide inhibition on day 3. Moreover, lactic acid, one of the degradation products of biodegradable PLGA, possesses intrinsic immunosuppressive and anti-inflammatory properties. The nanosphere-encapsulated alginate microbeads (control alginate microbeads) had 18.45 ± 1.56% nitric oxide inhibition on day 3 because the lactic acid degraded from the nanosphere-encapsulated alginate microbeads. Figure 9 shows the nitric oxide inhibition of murine macrophage cells by CsA in this system via a controlled drug delivery system. CsA was subsequently released to regulate the human immune system. As a result, CsA-encapsulated alginate microbeads are highly recommended for the delivery of anti-inflammatory and local immunosuppressive drugs to treat inflammatory diseases.

#### 3.2.7 Cell Viability Percentage of CsA Released from the Alginate Microbeads

**Figure 10:**
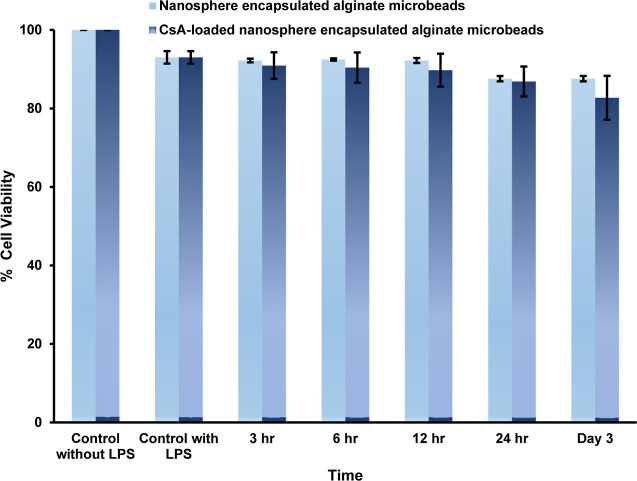
Cell viability percentage of nanosphere-encapsulated alginate microbeads and CsA-loaded nanosphere-encapsulated alginate microbeads on RAW 264.7 cells.

The MTT assay was used to assess the in vitro cell viability of the macrophage cell line (RAW 264.7) against the CsA released from the alginate microbeads. The controls for this investigation were RAW 264.7 cells treated with LPS and those not treated with it. The nanosphere-encapsulated alginate microbeads served as the control microbeads in this study, while the CsA-loaded nanosphere-encapsulated alginate microbeads were utilized as the study microbeads. Figure 10 shows a summary of the percentage of cell viability of the control and study microbeads. To examine cell viability, RAW cells were treated with CsA released from the sample microbeads and the released solution from control microbeads after 3 h, 6 h, 12 h, 24 h, and day 3. Figure 10 indicates that the released CsA from the sample microbeads showed 82.73 ± 5.58% cell viability on day 3. However, nanosphere-encapsulated alginate microbeads had a RAW cell viability of 87.59 ± 0.69% on day 3.^31,53−55^

## 4 Conclusions

In summary, a novel drug delivery system for treating inflammatory conditions was developed as CsA-loaded nanosphere-encapsulated alginate microbeads, which were used in this study to evaluate their controlled release profile, cell viability, and anti-inflammatory effects. Before encapsulation into alginate microbeads, CsA-nanospheres had a PDI of 0.517 ± 0.010, a 418.70 ± 59.08 nm diameter, and a zeta potential of −22 ± 0.57 mV. After the fabrication of CsA-loaded nanosphere-encapsulated alginate microbeads, the size of the microbeads was 635.75 ± 15.07 μm. Moreover, the CsA-loaded nanosphere-encapsulated alginate microbeads were more stable for 37 days than the nanosphere-encapsulated alginate microbeads. CsA was slowly released from the alginate microbeads via zero-order kinetics with no initial burst release within 24 h. In the drug release profile, the microbeads released 5.60% of the CsA within 24 h, and 85.90% of the drugs were diffused from the microbeads until day 64. However, the CsA released from the alginate microbead to treat acute liver failure is only required for 14 days.^12^ On day 3, the nitric oxide inhibition of the nanosphere-encapsulated alginate microbeads was 18.45 ± 1.56%, whereas CsA-loaded nanosphere encapsulated alginate microbeads exhibited 39.47 ± 1.71% nitric oxide inhibition on the RAW 264.7 cell. In comparison to the RAW 264.7 cell viability of nanosphere-encapsulated alginate microbeads with 87.59 ± 0.69% on day 3, the released CsA from the alginate microbeads had 82.73 ± 5.58% of cell viability on day 3. According to the anti-inflammatory and cell viability test, the CsA-loaded nanosphere-encapsulated alginate microbeads are effective in protecting the body’s immune system with a controlled drug delivery method. Based on this research design, the controlled release of CsA-loaded nanosphere-encapsulated alginate microbeads provides an innovative treatment for inflammatory diseases.
